# Genetic Dissection of Grain Size and Grain Number Trade-Offs in CIMMYT Wheat Germplasm

**DOI:** 10.1371/journal.pone.0118847

**Published:** 2015-03-16

**Authors:** Simon Griffiths, Luzie Wingen, Julian Pietragalla, Guillermo Garcia, Ahmed Hasan, Daniel Miralles, Daniel F. Calderini, Jignaben Bipinchandra Ankleshwaria, Michelle Leverington Waite, James Simmonds, John Snape, Matthew Reynolds

**Affiliations:** 1 John Innes Centre, Norwich research Park, Norwich, NR4 7UH, Norfolk, United Kingdom; 2 CIMMYT, Int. Apdo. Postal 6-641, 06600 México, DF, Mexico; 3 Cátedra de Cerealicultura, Departamento de Producción Vegetal, and IFEVA-CONICET, Facultad de Agronomía, Universidad de Buenos Aires, Av. San Martin 4453, C1417DSE Buenos Aires, Argentina; 4 Plant Production and Plant Protection Institute, Universidad Austral de Chile, Campus Isla Teja, Valdivia, Chile; Oklahoma State University, UNITED STATES

## Abstract

Grain weight (GW) and number per unit area of land (GN) are the primary components of grain yield in wheat. In segregating populations both yield components often show a negative correlation among themselves. Here we use a recombinant doubled haploid population of 105 individuals developed from the CIMMYT varieties Weebill and Bacanora to understand the relative contribution of these components to grain yield and their interaction with each other. Weebill was chosen for its high GW and Bacanora for high GN. The population was phenotyped in Mexico, Argentina, Chile and the UK. Two loci influencing grain yield were indicated on 1B and 7B after QTL analysis. Weebill contributed the increasing alleles. The 1B effect, which is probably caused by to the 1BL.1RS rye introgression in Bacanora, was a result of increased GN, whereas, the 7B QTL controls GW. We concluded that increased in GW from Weebill 7B allele is not accompanied by a significant reduction in grain number. The extent of the GW and GN trade-off is reduced. This makes this locus an attractive target for marker assisted selection to develop high yielding bold grain varieties like Weebill. AMMI analysis was used to show that the 7B Weebill allele appears to contribute to yield stability.

## Introduction

In many plant species there is a negative correlation between the number of seeds produced and the size of those seeds [1]. Genetic and physiological understanding of this trade-off is fundamental to increasing yield gains in crops for which the seed are the harvested part. Amongst these crops, wheat, rice and maize provide the staple foods of the world. For wheat, recent years have seen the rate of increase in production slow down, and the need for a new effort in the genetic improvement of this crop has been highlighted [2–4]. Up to now, genetic gains in grain yield potential of wheat have mainly been achieved by increasing grain number per unit area of land [5]. Breeders have selected varieties in which the extent of the grain weight trade-off is minimised. Generally, genetic gain for grain yield has been achieved as a product of stable or even reduced GW, but increased GN. This echoes a broader phenomenon for flowering plants in which GW displays low levels of phenotypic plasticity relative to GN, which is highly plastic in response to the environment and more genetically variable [1].

In spite of this general trend, it is possible to produce high yielding wheat varieties with large grains. Breeding strategies at the International Centre for the Improvement of Maize and Wheat (CIMMYT) have led to the development of a series of successful varieties widely adapted to target mega-environments (MEs) (Braun et al., 1996). Some CIMMYT varieties are notable in that they achieve high grain yields with a relatively large contribution from GW. For example, the CIMMYT bred varieties Baviacora and Kambara were identified as large grain types [6]. The characteristics of these varieties are known to be useful because the large grain trait, often referred to as bold seed, is preferred in many markets, improves the milling efficiency [7] and also aids the establishment of seedlings in stressed environments [8]. It is also the case that although historical progress in breeding is clearly associated with GN, GW is often associated with grain yield in the physiological assessment of contemporaneous varietal panels [9]. It is possible that other beneficial effects of the large grain trait in these varieties are not yet understood, but that multi-site selection for grain yield *per se* and grain yield stability has acted on this trait. Looking towards future grain yield gains, understanding the genetic and physiological basis of high GW in these varieties could facilitate the optimal expression of both yield components.

Here we present genetic and physiological analysis of a segregating population derived from two CIMMYT varieties, Weebill and Bacanora which are well adapted to similar environments but differ for the dominant grain yield components [10–11]. We present a single QTL that increases grain size without reducing grain number per unit area. This explains some of the large grain with high yield characteristics of Weebill. By growing the Weebill x Bacanora doubled haploid population at sites in Mexico, Argentina, Chile, and the UK, we show that this grain size QTL is very stably expressed and may contribute to yield stability across diverse environments in large grain varieties like Weebill.

## Results

### Grain yield in Weebill, Bacanora, and the WxB population

Yield data for Weebil, Bacanora, and WxB population means together with components of variance and trait heritabilities are shown in Table 1. The four years trial data in Mexico (Ob_07, Ob_08, Ob_09, Ob_10), two in the UK (CF_8 and CF_10), two in Chile (Va_09 and Va_10), and one in Argentina (BA_09) provided an environmental range that produced mean population grain yields from 5.5 t ha^−1^ (CF_10) to 12.2 t ha^−1^ (Va_10). The climatic data presented in Table A in S1 File goes some way to explaining this range. The low yield at CF_10 coincides with a very dry period over SEP and GFP (April to July) with rainfall 59% below the local average during these months in 2010. The rainfall for CF_08 is close to the local average. All non UK trials were irrigated so variability in rainfall is not likely to be a major factor explaining variation in grain yield for these experiments. The very high yields of Valdivia are accompanied by the highest rates of PAR at all growth stages, except for Va_09 at GFP for which Ob_10 recorded a higher figure, 10.6 and 12.4 MJ m-2 d-1 respectively. With the exception of one trial, BA_09, Weebill was the highest yielding parent in all environments. The positive difference was greatest in CF_10 where Weebill produced 31.3% more grain yield than Bacanora, and least in Ob_10, where the figure was 1.7%.

**Table 1:** Mean grain yield and its numerical components for Weebill, Bacanora and WxB doubled haploid population.

Trait	Env	Weebill	Bacanora	WxB mean	WxB sd	WxB CV	WxB her.block	WxB adj.r.sq	WxB Pr.F.	WxB signif
GRYLD	BA_09	6.6	7.0	6.9	1.32	19.3	0.6	0.35	4.27E-07	***
	CF_08	9.8	8.6	8.0	1.65	20.7	0.66	0.39	3.91E-11	***
	CF_10	6.3	4.8	5.5	1.03	18.7	0.92	0.79	1.51E-50	***
	Ob_07	6.4	6.1	5.9	0.85	14.5	0.76	0.4	8.84E-06	***
	Ob_08	NA	NA	7.1	0.92	13	0.75	0.6	1.54E-12	***
	Ob_09	7.2	6.1	6.3	0.89	14.2	0.85	0.7	4.35E-17	***
	Ob_10	5.9	5.8	5.8	0.76	13	0.17	0.09	0.172	
	Va_09	12.9	11.7	12.1	2.15	17.7	0.7	0.44	1.55E-14	***
	Va_10	12.7	11.8	11.9	2.2	18.4	0.58	0.32	2.80E-08	***
GRPSQM	BA_09	13939.5	25484.8	21800	4210	19.4	0.45	0.24	0.000479	***
	CF_08	NA	NA	16900	3600	21.4	0.64	0.39	3.68E-10	***
	CF_10	15991.5	13374	13400	2750	20.4	0.91	0.77	1.93E-47	***
	Ob_07	12864.9	13048	15648	2700	17.2	0.81	0.49	3.72E-08	***
	Ob_08	NA	NA	18432	3160	17.2	0.87	0.76	6.18E-23	***
	Ob_09	12328.67	17908.5	16750	2720	16.2	0.86	0.69	8.10E-17	***
	Ob_10	28410.2	14973.5	14900	2380	16	0.57	0.4	9.70E-06	***
	Va_09	27645.2	30143.7	27300	5170	18.9	0.75	0.5	2.99E-18	***
	Va_10	27326.6	30719.3	26800	4960	18.5	0.83	0.62	2.06E-28	***
TGRWT	BA_09	38.1	27.3	32	4.75	14.8	0.91	0.79	3.75E-37	***
	CF_08	NA	NA	47.5	4.65	9.8	0.8	0.58	1.16E-21	***
	CF_10	50.12	35.77	41.1	3.88	9.5	0.87	0.69	3.82E-34	***
	Ob_07	40.13	36.22	37.9	4.64	12.2	0.89	0.79	9.04E-25	***
	Ob_08	NA	NA	38.7	3.68	9.5	0.85	0.72	4.41E-19	***
	Ob_09	45.5	34.13	37.7	3.75	10	0.91	0.83	2.70E-29	***
	Ob_10	46	38.8	39.5	3.34	8.4	0.9	0.82	3.69E-27	***
	Va_09	45.4	39	44.7	4.1	9.2	0.94	0.83	1.77E-63	***
	Va_10	46	38.7	44.2	4.24	9.6	0.95	0.87	7.05E-73	***

ANOVA and heritabilities are for the WxB population. Abbreviations are: mean, unadjusted arithmetic mean; var, variance; sd, standard deviation; CV, coefficient of variation; her. Block; heritability estimate; r.sq; R-squared; adj.r.sq, adjusted R squared; Pr.F, p value of F test; signif, significance level (***0.0001, ** 0.001, * 0.05; F, F statistic; df, degrees of freedom; df.res; df in the residuals; sum.sq; sums of square for trait; sum.sq.res, sums of square for residuals; mean.sq, means of square for trait; mean.sq.res, means of square for residuals; anovamean, mean from the linear model (or anova); ranef.se, standard errors of random effects; site, site; year. NA indicates that data was not available. Equivalent data for DTAD, DTEM, and HT are shown in Table D in S1 file.

F, F statistic; df, degrees of freedom; df.res; df in the residuals; sum.sq; sums of square for trait; sum.sq.res, sums of square for residuals; mean.sq, means of square for trait; mean.sq.res, means of square for residuals; anovamean, mean from the linear model (or anova); ranef.se, standard errors of random effects; site, site; year. NA indicates that data was not available. Equivalent data for DTAD, DTEM, and HT are shown in Table D in S1 File.

**Fig 1:**
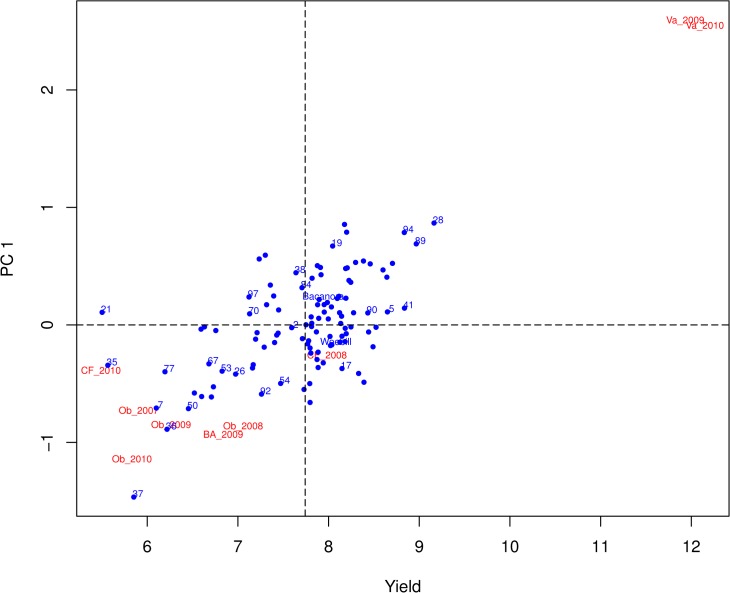
AMMI 1 plot of grain yield for Weebill, Bacanora, and WxB segregating population. See Tables A and B in S1 File for full descriptions of environments. Yield is the mean of all environments shown. Genotype close to 0 for PC1 (Principal component 1) show low levels of environmental interaction for this trait. Proximity to an environment data point indicates that the genotype in question is relatively well adapted to that environment.

**Fig 2:**
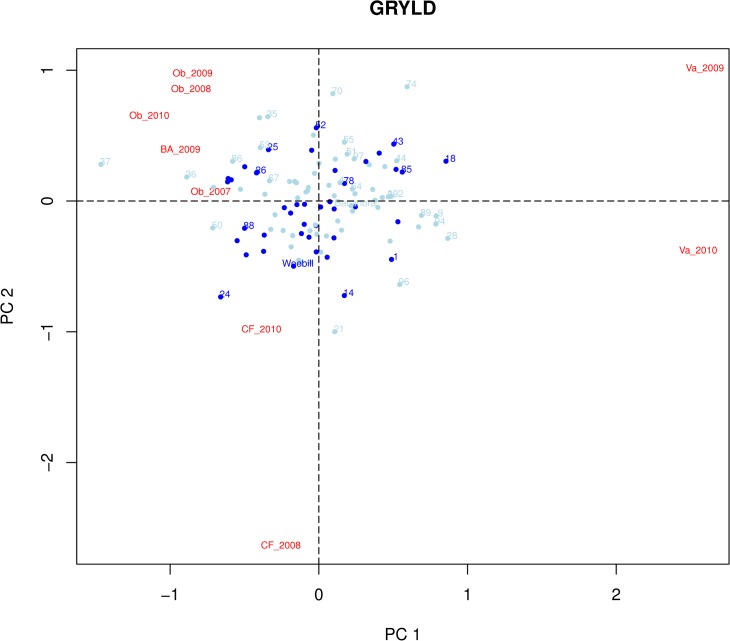
AMMI 2 plot. Principal components 1 and 2 from AMMI analysis of grain yield are plotted against each other. Genotypes closest to zero for PC1 and PC2 show the highest levels of yield stability. Genotypes carrying the Weebill (increasing) allele of the 7B grain size QTL are shown in dark blue.

Genotype x Environment interactions for GRYLD were investigated further using AMMI (Additive Main effect Multiplicative Interaction) analysis [12]. AMMI1 and AMMI2 biplots [13] are shown in Fig. 1 and 2. Both plots show that the two parents display low levels of interaction with the environment (close to zero for Principle component 1 and 2). A number of segregants, such as WB05, WB90, and WB41 showed consistent transgressive segregation for GRYLD combined with yield stability (low PC1 and PC2 values) equal to the elite parental varieties. Other genotypes also showed high average yield but with stronger environmental interaction. So WB94 gave the same mean GRYLD as WB41 but with less stability. The Chilean trial site, Valdivia, proved to be the highest yield potential environment for this population in both years. The lines WB28, WB89, and WB94 had highest average yield but were specifically adapted to the very high yield potential environment of Valdivia.

### Components of grain yield in Weebil x Bacanora

The majority of traits were quantitatively inherited. However, this was not the case for heading date in Ob_07,Ob_08, Ob_09, or Va_08, Va_09. This is because the two parents carry different dominant genes conferring spring habit so one quarter of the segregants are winter types. In these years vernalization requirement was not fully satisfied so heading date was not normally distributed. Correlation of heading date with GRYLD, GRpsqm, and TGRWT is shown in Table 2. There was significant positive correlation of heading date with GRYLD in five out of nine environments. In all cases this was driven by positive GRpssqm and also occurred when vernalization requirement was fully satisfied, as in CF_10. In terms of TGRWT, the main focus of this study, there was a highly significant negative correlation in Ob_09 (−0.36, p<0.0010) and less significant negative correlations (−0.23 to 0.27, p<0.05 threshold) in Va_09, Va_08, and Ob_07. There is no indication from this data that increased grain size without concomitant trade off in grain number is realted to variation in heading date in W x B.

**Table 2:** Correlation of GRYLD, GRpsqm and TGRWT with heading date.

	GRYLD	GRpsqm	TGRWT
BA_09	0.00	-0.08	0.05
CF_08	0.13	0.30**	−0.28*
CF_10	0.55***	0.59***	−0.10
Ob_07	0.32**	0.42***	−0.24*
Ob_08	0.24*	0.21	−0.05
Ob_09	0.28**	0.46***	−0.36***
Ob_10	0.08	-0.05	0.13
Va_08	0.23*	0.33**	−0.24*
Va_09	0.16	0.27*	−0.23*

Asterisks denote significance levels of correlations

0.05 (*)

0.01 (**)

0.001 (***)

Analyses of variance (ANOVA) showed significant genotypic effect on all traits measured, this analysis is shown in Table 1. The TGRWT and GRpsqm data validated the selection of these parents to dissect grain number/size relationships in bread wheat. Bacanora produces more grain per unit area than Weebill (between 6% and 32% greater) in all environments, except Ob_07 (7% less) but Weebill consistently has a greater TGRWT than Bacanora (between 10% and 40% greater).

To gain insight into how other traits fit into the relationship of GRpsqm and TGRWT with GRYLD in WxB, the correlation of all traits with grain yield was analysed. Correlation of GRYLD with TGRWT and GRpsqm are shown in Table 3. In all environments the strongest positive correlation with GRYLD was GRpsqm. TGRWT was not correlated with grain yield, except for BA_09 where both yield components explained GRYLD similarly.

**Table 3:** Correlation coefficients of thousand grain weight (TGRWT) and grains per unit area of land (GRpsqm) with grain yield (GRYLD).

Env	TGRWT	GRpsqm
Va09	0.13	0.90***
CF08	0.22*	0.90***
Va08	0.08	0.86***
Ob10	−0.11	0.84***
BA09	0.55***	0.75***
Ob08	−0.29**	0.76***
Ob09	0.07	0.78***
Ob07	0.05	0.87***
CF10	0.16	0.56***

Asterisks denote significance levels of correlations

0.05 (*)

0.01 (**)

0.001 (***)

The correlations of GRpsqm with its numerical components are shown in Table 4. All were positively correlated with GRpsqm). Across environments, grain number per spike (GRNBpSP) exhibits the highest correlation coefficients, suggesting that this is a relatively stable determinant of grain number in diverse environments and across different sowing rates adopted at each environment. The data provides some evidence of buffering between these related traits. Thus, in Ob_10, GRNBpSP shows a relatively low and non significant correlation with GRNpsqm (0.139), but it is in this environment that the correlation with spike number per square metre (SPNBpsqm) was the highest (0.52). The trait that showed some of the highest correlation with GRNpsqm, particularly in the environments with high yield potential, was above ground biomass (PLBM). When significant this ranged from 0.43 atCF_10 to 0.756 in Va_09. In all but two cases, BA_09 and Ob_10, Ht is also associated with increases in GRNpsqm the highest value being 0.6 from CF_10, but no significant correlation in BA_09 or Ob_10.

**Table 4:** Correlation coefficients of the determinants of grain number and biomass traits with grains per unit area of land (GRpsqm), full trait names are given in Methods, Env is environment, full environment names are in Table C in S1 File.

Env	GRNBpSPT	GRNBpSP	SPTNBpEAR	SPNBpsqm	PLBM	HI	Ht
BA09	0.31**	0.48***	0.35**	0.60*** ^s^	0.66***	0.14	-0.16
CF10	0.54***	0.68***	0.50***	NA	0.43***	0.76***	0.57***
Ob07	0.61***	0.70***	0.27*	0.44***	NA	NA	0.26*
Ob08	0.59***	0.66***	0.28**	0.38***	-0.14	0.69***	0.23*
Ob09	NA	NA	0.32**	NA	0.11	0.42***	0.37***
Ob10	-0.01	0.14	0.32**	0.74***	0.74***	-0.22*	0.14
Va08	NA	0.91***	NA	0.49***	0.75***	0.65***	0.34**
Va09	NA	0.90***	NA	0.42***	0.76***	0.64***	0.25*

Asterisks denote significance levels of correlations

0.05 (*)

0.01 (**)

0.001 (***)

### Grain yield QTL and coincident yield component QTL

**Fig 3:**
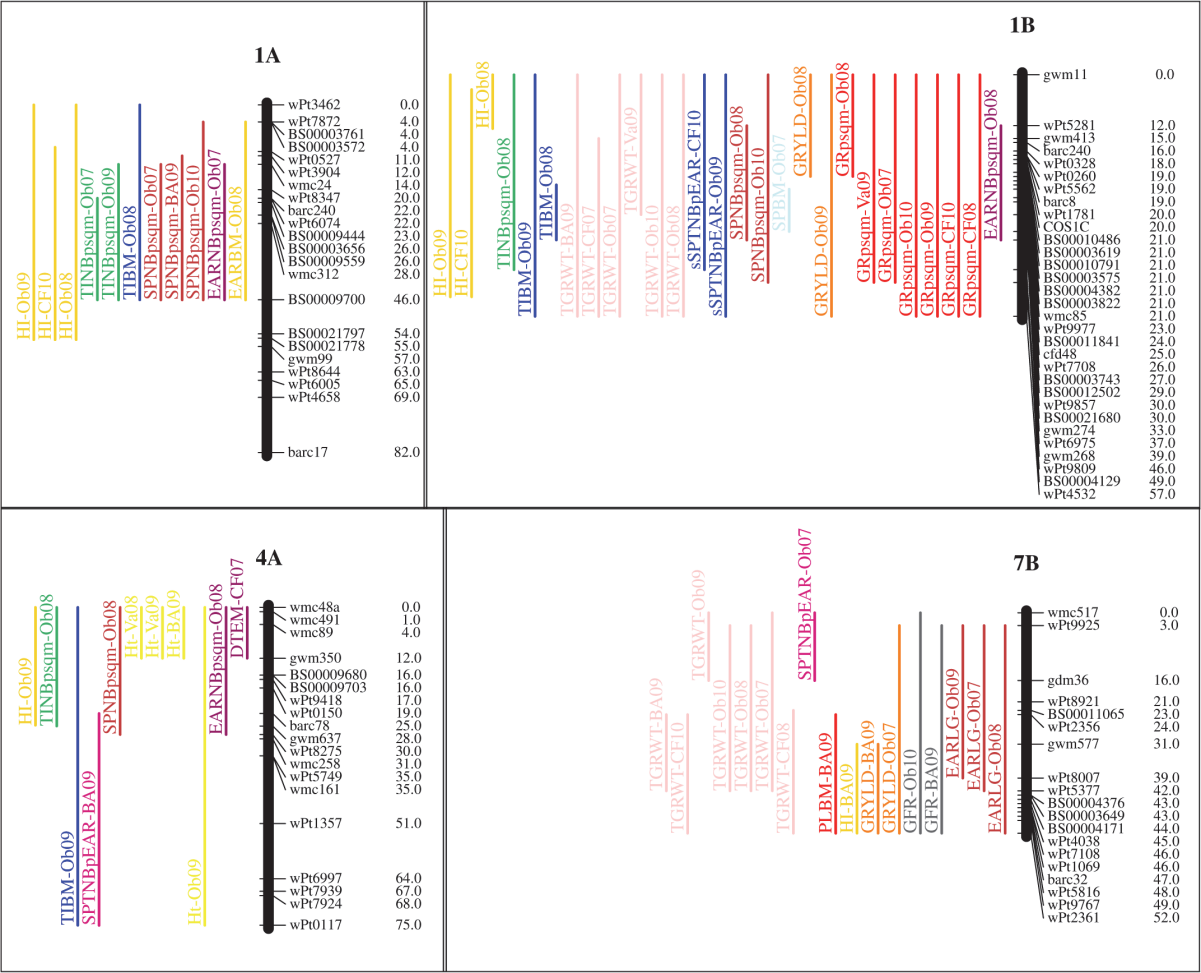
Chromosomal locations of QTL identified in Weebill x Bacanora. Chromosomes carrying QTL for the same trait identified in more than two environments are shown. Coloured bars represent QTL confidence interval. Shortened trait names are given in methods. Trait names are appended with names of environments in which they were detected (see Table C in S1 File).

Table 5 shows QTL identified through independent analysis of data from each environment, where growth habit was segregating a number of pleiotropic effects cosegregated with *Vrn-B1*, these are not the focus of this study and are not included in Table 5. All significant QTL identified together with more information on genetic location are shown in Table B in S1 File. Fig. 3 shows the chromosomal location of QTL identified in more than three environments. As the focus of this analysis is the relationship between grain size, grain number, and grain yield the description of effects will begin with cases in which allelic variation for yield components was detectable as a yield effect, while bearing in mind the relatively low heritability of grain yield compared to yield component traits and the QTL detection power provided by a population of 105 doubled haploid individuals. QTL for GRYLD were identified in four of the nine environments sampled. They were on 1B in Ob_08, Ob_09 and 7B in Ob_07 and BA_09. In all cases the yield increasing allele was from Weebill with additive effects of 0.41, 0.23, 0.23, and 0.58 t ha^−1^, respectively.

**Table 5:** QTL with a LOD score greater than 2 excluding those that collocated with *Vrn-B1*.

Trait	Chromosome	LOD	Variance explained (%)	Mean	Additive effect	Environment
GRYLD	1B	2.2	11.2	6.29	−0.23	Ob_09
GRYLD	1B	3.8	15.8	7.089	−0.41	Ob_08
GRYLD	7B	2.3	11.6	5.901	−0.23	Ob_07
GRYLD	7B	4.7	22.7	6.771	−0.58	BA_09
GRpsqm	1B	2.2	11.4	16653	−1008	CF_08
GRpsqm	1B	2.6	13.8	13325	−930	CF_10
GRpsqm	1B	2.7	11.8	16877	−989	Ob_09
GRpsqm	1B	2.9	12.4	14962	−729	Ob_10
GRpsqm	1B	3.1	15.2	15777	−929	Ob_07
GRpsqm	1B	3.4	16.6	27125	−1805	Va_09
GRpsqm	1B	5.4	22.2	18630	−1629	Ob_08
TGRWT	1B	2.1	8.2	38.447	1.36	Ob_08
TGRWT	1B	2.2	7.8	39.332	1	Ob_10
TGRWT	1B	2.3	10.2	44.705	1.5	Va_09
TGRWT	1B	2.9	12.5	37.748	1.38	Ob_07
TGRWT	1B	3.2	12.8	31.909	2.02	BA_09
TGRWT	7Aa	3	11	39.332	1.1	Ob_10
TGRWT	7B	2.8	12.6	47.313	−1.59	CF_08
TGRWT	7B	3.2	13.6	37.748	−1.55	Ob_07
TGRWT	7B	3.4	13.8	38.447	−1.45	Ob_08
TGRWT	7B	3.6	13.5	39.332	−1.45	Ob_10
TGRWT	7B	5	23.3	37.598	−1.6	Ob_09
TGRWT	7B	5.1	18.7	41.017	−1.65	CF_10
TGRWT	7B	5.2	22.1	31.909	−2.36	BA_09
SPNBpsqm	1A	2	8.7	249.53	12	Ob_10
SPNBpsqm	1A	3.4	16.2	518.83	27	BA_09
SPNBpsqm	1A	4	16.2	306.95	14	Ob_07
SPNBpsqm	1B	2.4	10.3	249.53	−13	Ob_10
SPNBpsqm	1B	4.7	15	374.35	−21	Ob_08
SPNBpsqm	2Ab	4.1	20.3	518.83	−29	BA_09
SPNBpsqm	4A	2.9	9.1	374.35	−15	Ob_08
SPTNBpEAR	2Bb	3.4	14.3	23.649	0.55	Ob_08
SPTNBpEAR	2D	2.2	11.6	16.733	0.29	CF_10
SPTNBpEAR	4A	2.7	13.7	17.836	0.42	BA_09
SPTNBpEAR	5Ab	3.1	13.2	20.048	−0.58	Ob_07
SPTNBpEAR	7B	2.2	8.9	20.048	0.49	Ob_07
GRNBpSPT	2Ab	2.3	14.7	2.358	0.099	BA_09
GRpSPT	2Aa	2.1	11.4	2.51	0.124	CF_10
TINBpsqm	1A	4.5	16.6	160.9	11	Ob_09
TINBpsqm	1A	4.8	22.5	376.36	20	Ob_07
TINBpsqm	1B	4.3	16	392.3	−21	Ob_08
TINBpsqm	4A	2.2	7.8	392.3	−15	Ob_08
sSPTNBpEAR	1B	2.2	11.1	2.004	0.169	Ob_09
sSPTNBpEAR	1B	4.1	20.5	0.872	0.344	CF_10
EARBM	1A	2.9	14.3	0.855	−0.054	Ob_08
TIBM	1A	2.1	8.5	2.46	−0.11	Ob_08
TIBM	1B	2.7	11.2	2.46	0.121	Ob_08
TIBM	1B	2.8	9.8	2.138	0.109	Ob_09
TIBM	4A	2.8	9.8	2.138	0.107	Ob_09
PLBM	7B	2.7	16.7	1599.4	−79.85	BA_09
SPBM	1B	4.7	17.4	9.327	1.104	Ob_07
HI	1A	3.1	12.9	0.163	0.011	CF_10
HI	1A	2.6	8.6	0.292	0.014	Ob_08
HI	1A	2.5	8.1	0.299	0.021	Ob_09
HI	1B	3.2	13.4	0.163	−0.011	CF_10
HI	1B	6.4	23.7	0.292	−0.027	Ob_08
HI	1B	5.5	19.2	0.299	−0.021	Ob_09
HI	2D	2	10.2	0.481	0.011	Va_09
HI	4A	2.5	8.1	0.299	−0.015	Ob_09
HI	7B	4.5	25.9	0.425	−0.015	BA_09
GFR	7B	2	12.6	0.92	−0.055	BA_09
GFR	7B	2.35	11.5	1.098	−0.028	Ob_10
EARLG	1Db	2	7.7	12.118	0.39	Ob_08
EARLG	3Aa	2	8.6	9.761	−0.24	CF_10
EARLG	5Ab	2.3	9	11.32	−0.3	Ob_09
EARLG	5Ab	2.8	11.5	11.586	−0.3	Ob_07
EARLG	7B	2.7	10.7	12.118	−0.38	Ob_08
EARLG	7B	3.9	16.7	11.586	−0.41	Ob_07
EARLG	7B	4.7	19.8	11.32	−0.46	Ob_09
EARNBpsqm	1A	3.9	15.9	153.67	7	Ob_07
EARNBpsqm	1B	5.1	17	374.35	−21	Ob_08
EARNBpsqm	4A	2.5	7.8	374.35	−14	Ob_08
Ht	3Aa	2.3	8.6	76.182	1.8	Va_09
Ht	4A	2.53	12.3	96.895	−1.8	Ob_09
Ht	4A	2.88	17.4	75.301	2.3	BA_09
Ht	4A	3.2	12.1	76.182	2.1	Va_09
Ht	4A	4.96	22.8	82.152	2.2	Va_08
Ht	6A	2.2	10	83.759	1.4	Ob_10
DTMA	3Aa	2.7	13.8	122.51	−0.07	CF_10

Chromosomes broken into multiple linkage groups carry a single letter suffix.

As much as possible, QTL analysis of data for the primary yield components of TGRWT and GRpsqm, were used to show the extent to which each of these trait components contributed to the yield effects identified. The most likely yield component effects underlying the 1B GRYLD QTL detected in Ob_08 and Ob_09 were QTL for increasing grain number (GRpsqm) with additive effects of 1629 to 989 grains per m^2^ from Weebill in the same year and locations. The relatively low QTL detection power provided by 105 individuals of the Weebill x Bacanora population means that we cannot say how likely the Ob_08 and Ob_09 QTL are to be the same, in that they are caused by the same polymorphism. However, the Ob_08 1B GRpsqm QTL is detected together with a SPNBpsqm increasing allele from Weebill with an additive effect of 21 spikes per square metre. This suggests that the basis of the yield effect could be increased spike production in this case. Unfortunately, SPNBpsqm data for Ob_09 was not available so the basis of increased grain number cannot be commented on for the Ob_09 1B GRYLD QTL. Taking into consideration the question of locus specific GW and GN trade off, it can be seen that in Ob_08 there was a coincident 1B effect of reduced TGRWT, with the decreasing allele from Weebill carrying an additive effect of 1.36 g.

The 7B yield QTLs detected in Ob_07 and BA_09 are not associated with positive GN effects but are located in the same region as QTL for which with Weebill alleles increase TGRWT with an additive effect of 1.55 g and 2.36g, respectively. Considering GW and GN trade off in this case, it can be seen that no increasing effects for GRpsqm are detected from Bacanora alleles at this locus.

### Primary yield component QTL not coincident with 1B and 7B yield QTL

In addition to the two GRpsqm QTL on 1B that are coincident with yield effects (Ob_08 and Ob_09), the positive effect on GRpsqm of Weebill alleles at this locus was also detected in Va_08, Va_09, Ob_07, and Ob_10. In all of these experiments, apart from Va_08, a coincident 1B QTL for TGRWT was identified with the decreasing allele from Weebill, so preventing the increase in GN from being expressed as GRYLD. In spite of the fact that the main driver of GRYLD in the populations as a whole is GRpsqm, the potential of the 1B GRpsqm QTL to manifest itself as a GRYLD QTL is limited, in most of the environments sampled, by the negative trade off effect of increased GRpsqm on TGRWT.

The increasing effect on TGRWT from Weebill on 7B, like 1B, is found together with GRYLD QTL in two environments (Ob_07 and BA_09). It was also detected as an effect on TGRWT without yield in Ob_08, Ob_10, and CF_10 with additive effects ranging from 1.5 to 1.7g of TGRWT, but in these cases with no detectable effect on GRYLD. This QTL was not collocated with a depressive effect on GRpsqm from Weebill in any of the environments tested. So, although the increasing effect of the 7B Weebill allele is not always translated into grain yield, it does appear that Weebill alleles at this locus have the potential to confer larger grain size with a very limited trade-off for GN. The relatively consistent TGRWT QTL detected on 7B might not always be identified as GRYLD QTL because of the lower heritability of grain yield (Table 1) and some reduction in GRpsqm that was not identified as a significant QTL.

### QTL for components of grain number

In this study the trait most associated with grain yield at the whole population level was GRpsqm. At the whole population level, the components of GRpsqm most consistently correlated with grain yield were grain number per spike (GRNBpSP) and spike number per unit land area (SPNBpsqm). However, no QTL for GRNBpSP were identified. There are seven QTL identified for SPNBpsqm. On 1A, QTL for SPNBpsqm, with increasing effects from Bacanora, were identified in Ob_07, Ob_10, and BA_09 with additive effects of 12–27 spikes per square meter. QTL for SPNBpsqm with increasing effects from Weebill were identified on 1B in Ob_08 and Ob_10 with additive effects of 21 and 13 spikes per square meter. Single effects were also found in BA_09 on 2A and in Ob_08 on 4A.

### Other QTL associated with 1B and 7B yield effects

Loci at 1B and 7B emerged as QTL ‘hotspots’ in the analysis of Weebill x Bacanora data, see Fig. 3. This included traits other than the direct components of grain yield. At the whole population level, biomass traits showed high correlations with GRYLD confirming previous observations in the WxB population [10–11]. The most frequently detected biomass effects were on 1B. Bacanora alleles on 1B that increased tiller biomass TBM in Ob_07, Ob_08, and Ob_09, final spike biomass (SPBM) in Ob_07, and final plant biomass (PLBM) in Ob_07. For 7B the BA_09 yield QTL is accompanied by a Weebill increasing effect for PLBM.

The rate of grain filling was measured in Argentina, Chile and Mexico. In BA_09 and Va_09, Weebill alleles increased the rate of grain fill with additive effects of 55 and 28 mg d^−1^. There seem to be multiple effects on inflorescence growth as a whole, as another pleiotropic or linked effect for the 7B QTL is an increase in spike length from Weebill alleles. This was observed in three of the four environments in which it was measured, Ob_07, Ob_08, and Ob_09, with a full substitution effect of 7.6–9.2mm.

### Other QTL for agronomic traits

For crop height six QTL with additive effects between 1.4 and 3 cm were identified. Three of these are specific to single environments (3A in Va_09, 4A in Ob_09, and 6A in Ob_10), but 4A effects (independent from the Ob_09 QTL) were detected in BA_09, Va_08, and Va_09 with the increasing allele from Bacanora. For ear emergence one major effect was identified at *Vrn-B1* when vernalization requirement was not satisfied (data not shown). The spring habit gene which Bacanora carries was not identified, but the genetic map did include coverage of *Vrn-1* regions of 5D and 5A.

### The effect of 1B and 7B QTL on yield stability

**Fig 4:**
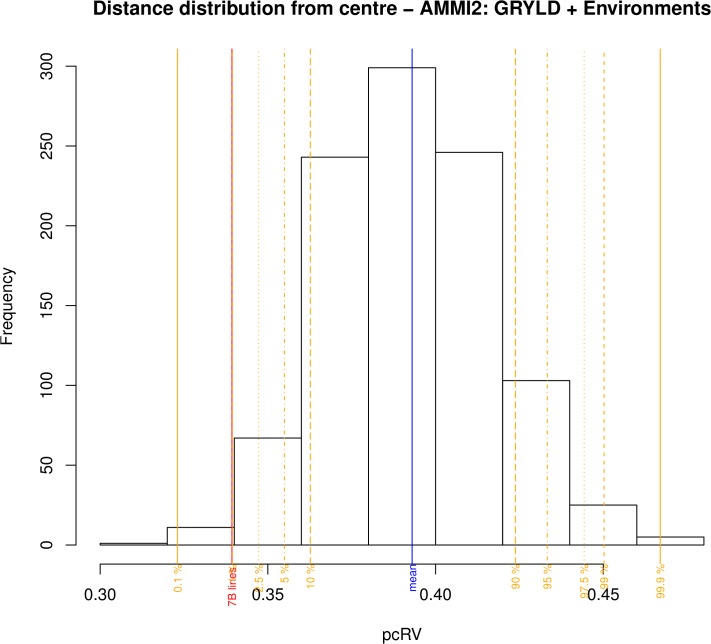
Estimating the effect of 7B grain yield QTL on yield stability. The distribution represents the frequency of summed distances from the point of AMMI2 plots where PC1 and PC2 are equal to zero in 1000 random permutations for the number of lines carrying the Weebill allele of the 7B GRYL QTL described in the text. The summed distances for actual data set (dark blue circles in Fig. 2) occurred in less than 2% of the simulated data sets, marked with vertical red line labelled 7B.

In an attempt to test whether the GRYLD QTL identified in this study might have any effects on adaptation we used AMMI statistics to calculate the effects of QTL on yield stability. In Fig. 2, WxB lines carrying the Weebill allele of the 7B TGRWT/GRYLD effect are shown in dark blue. Fig. 4 shows that the lines carrying the Weebill allele were significantly closer (see methods section for calculation of significance) to a PC1 and PC2 value of zero than those carrying the Bacanora allele, and therefore displayed higher levels of yield stability. When the same process was repeated for the 1B GRYLD QTL, there was no significant clustering of either allele towards the centre of the AMMI2 plot.

## Discussion

Through the process of wheat breeding, direct selection for grain yield *per se* mostly results in an increase in grains per unit area, because this is by far the most plastic of the two yield components [1]. However, maximum theoretical yield potential require a minimisation of the resultant GW trade-off. Here we confirm that progeny of a cross between varieties with similar yield potential but differing in the balance of yield components results in extensive transgressive segregation for grain yield and yield stability as was described in the same population by [10–11]. Our data confirms the findings of most studies of yield progress in bread wheat, that grains per unit area is the component most closely correlated with grain yield [5]. However, the highest yielding segregants in the WxB population are those that show the greatest positive deviation from the negative correlation of grain size and number. This is a breeding truism, the highest yielding segregants are those with the most grains and the biggest. The work described here sheds some light on specific genetic variation that can be deployed to achieve this.

Two grain yield QTL were identified and analysed in the context of GW and GN trade-off. The effect on chromosome 1B could be a consequence of the segregation of the 1RS rye translocation in this population. The 1BL.1RS translocation was introduced into CIMMYT germplasm through Veery lines based on crosses with North American germplasm. This clearly introduced novel disease resistance, but effects on grain yield have been less clear with positive and negative effects seen in a series of studies [14–18], presumably due to interactions with environment and genetic background. In this study, and despite the positive effect on biomass detected in some environments, WxB segregants carrying the translocation generally display lower grain yield and no crossover effects were seen between the environments sampled. If the 1B effects identified in this study are due to 1BL.1RS our data suggests that, where 1BL.1RS has shown beneficial yield effects, interactions not encountered in the experiments described here allowed the conversion of this biomass to grain.

The second yield QTL identified on chromosome 7B was of particular interest to us. We propose that this yield QTL was caused by a very stably expressed GW QTL which is not coincident with any reduction in GN. This describes the type of effect that would allow the development of large grain varieties like Weebill, and a positive deviation from GW and GN trade off. Although QTL analysis for this deviation was undertaken no significant effects were identified. This may be because this trait is mathematically derived from directly measured traits for which compounded variances cause a loss of QTL detection power as described previously [19]. From analysis of single trait QTL, we can state that the substitution effect of the Weebill allele at the 7B locus amounts to a mean increase in GW of 3.4 mg. In all environments there was no concomitant decrease in GN, and in two of those environments the effect on GW is detected as a yield QTL. This included BA_09, the only environment in which grain size was highly correlated with grain yield.

The nature of the 7B grain size QTL allows us to speculate that it may contributes to yield stability by providing lines with a wider repertoire of outlets for yield potential in environments where GW is important, but not carrying a penalty where it is less so. This is supported by AMMI2 analysis showing that lines carrying Weebill alleles at this locus do display increased levels of yield stability compared to those carrying Bacanora alleles. It appears that it is not simply the case that there is a developmental and morphological limit to grain number after which grain size comes into play. The highest GN occurred in Valdivia, where the 7B QTL was not detected as a yield effect. The ability to manipulate grain size independently of number provides strategies to reduce yield losses associated with stress during the rapid spike growth phase [20] as increased grain size is the only route to some recovery in yield potential after losses during this phase.

In spite of numerous studies reporting the location of grain size QTL in bread wheat [21–27] there are no reports of any grain yield QTL in which the increasing yield component is grain size associated with the region of 7B reported here. The multiple environment trials and QTL analysis described here shows that the effect is very stably expressed. Weebill alleles at the 7B locus also increase spike length. Previous selection experiments for GW showed this as a general relationship [28].

This study sheds some light on how the grain size QTL described influence the physiological determinants of grain weight, and why the Weebill allele of the 7B QTL influences grain size relatively independently of grain number. Variation on grain size can be achieved through traits expressed before and after anthesis [29]. In the present work the variation in grain size was associated with changes in the rate of grain filling. The two environments in which the 7B locus is detected as a yield effect are BA_09 and Ob_07. It is for BA_09 that we repeat the findings of previous studies {Lopes, 2012 #887} that TGRWT can be become a more important driver of yield in certain environments. It is notable that the lowest rates of PAR were recorded in BA_09, across all growth stages. In spite of this yields in BA_09 were by no means the lowest, ranked fifth out of nine environments. It is possible that post anthesis grain filling compensated for these relatively low light levels that were experienced during pre anthesis grain number determination. Some extra weight is added to this suggestion by the observation that in also Ob_07 the PAR levels are relatively low, out of the seven environments where PAR was measured: third lowest at LSP, second lowest at both SEP and GFP. It is possible that the yield QTL is being expressed in these environments because it allows the crop to compensate for low GN during grain filling.

Future work will facilitate the detailed physiological and developmental analysis of this effect as well as the genetic dissection of the locus to identify the gene/s underlying the QTL. To this end Near Isogenic Lines for the 7B QTL are now being developed.

## Materials and Methods

### Weebill x Bacanora DH recombinant population

The Weebill x Bacanora (WxB) population comprises 105 recombinant doubled haploid lines derived from F_1_ using the maize cross method [30]. The parents are *RhtB1b* semi-dwarfs and carry the photoperiod insensitive *Ppd-D1a* allele of *Ppd-D1*. Bacanora also carries the 1BL.1RS rye translocation. For Weebill, spring habit is conferred by *Vrn-B1a*. The genetic basis of spring habit in Bacanora is not known.

### Environments

Locations and years in which the WxB population, parental lines were grown, and experimental designs are shown in Table C in S1 File. Climatic factors for these environments are described in Table A in S1 File and more detail of specific sites can be found in [11]. Best local agronomic practice was applied in each environment and year. In the UK (CF_10) and Chilean (Va_08 and Va_09) trials a complete randomised block was used, with three replicates. In Buenos Aires the trial was completely randomised with three replicates. In Mexico (Ob_07, Ob_08, Ob_09, and Ob_10 and alpha lattice with two replicates. Plot areas were: UK, 6m^2^; Mexico, 2m^2^, Chile 1m^2^, and Argentina 2m^2^. Seed density between 200–350 per m^2^ depending best local agronomic practice.

### Traits

The way in which raw phenotypic data was collected and physiological traits derived is now described. Ear emergence (EM) was recorded as the date upon half of the length of the ear is extended beyond the flag leaf ligule for half of all the stems in a plot. Days to ear emergence (DTEM) is the number of days between sowing date and EM. This is the same as GS55 on the Zadoks scale [31]. Maturity (MA) is the date on which the peduncle is fully senesced so that the ear no longer has access to water and nutrients from the stem. Days to maturity (DTMA) is the number of days between sowing and MA. This is the same as Zadoks GS 87. Ear biomass (EARBM) was measured as the mass of intact wheat ears detached from the stem at the collar. Ear biomass per square meter is the sum of EARBM collected from a one square metre quadrat or in some cases samples from a set row length which is multiplied to represent 1m^2^. Ear number per square meter (EARNBpsqm) is the number of those ears. Ear length (EARLG) is the length of the ear from the collar to the tip of the terminal spikelet, not including awns. Thousand grain weight (TGRWT) is the weight of 1000 wheat grains. Grain filling rate (GFR) was determined by dividing TGRWT the number of days from anthesis to maturity. Grain weight per spikelet was determined by removing grains from individual spikelets from a sample of twenty wheat ears and weighing the grain. Grain per spikelet (GRpSPT) was estimated by counting the number of grain set within each spikelet from the same sample as EARBM. Spike number per square meter (SPNBpsqm) was determined by counting spikes within a 1m^2^ quadrat or from a row within a plot of defined length. Grain per square metre (GRpsqm) was determined in two ways; by multiplying SPNBpsqm by the number of grains per spike; and by dividing grain yield per square metre by mean grain weight. Grain yield (GRYLD) refers to whole plot yield taken using plot combined harvesters, adjusted for moisture content, and divided by plot size to give grain yield per square metre. Plant biomass (PLBM) is the weight per square metre of all above ground crop ate maturity. Harvest Index (HI) is the proportion of PLBM that is grain. Spike biomass (SPBM) was calculated by weighing twenty detached spikes from a quadrat or defined row length at maturity. We arrived at Spikelet number per ear (SPTNBpEAR) by counting spikelets on the sample of twenty ears per plot. The non-seed bearing spikelets were also counted (sSPTNBpEAR). Tiller number per square metre (TINBpsqm) was based on quadrat or defined row length samples. Tiller biomass (TIBM) was arrived at by dividing PLBM by TINBpsqm.

### Genetic map construction

A framework genetic map was primarily developed using publically available Single Sequence Repeat (SSR) markers as anchors integrated with data from Diversity Arrays Technology Genome Profiling (DArT) by Triticarte. DNA of the population was sent to Triticarte Pty Ltd, Australia for analysis (http://www.triticarte.com.au/). SSR primer sets were used from JIC (psp), IPK Gatersleben (gwm/gdm), Wheat Microsatellite Consortium (wmc), Beltsville Agricultural Research Station (barc) and INRA (cfd/cfa) (for more information see GrainGenes website, http://www.wheat.pw.usda.gov/). SSR DNA fragments were amplified with PCR, run on 6% polyacrylamide gels for separation, and visualised using silver staining. SSR markers were used to orientate linkage groups using published Consensus maps (Somers et al. 2004) our aim being a density of one marker every 10 cM. In 2012, to further improve map coverage and take advantage of SNP marker technologies, publically available markers developed by the University of Bristol were added to the map using KASP (Competitive Allele-Specific polymerase chain reaction assay) developed by LGC Genomics. All primer information and protocols for the high throughput Genotyping Procedure can be found at CerealsDB (http://www.cerealsdb.uk.net/cerealgenomics/CerealsDB/kasp_mapped_snps.php).

Joinmap v3.0 was used for genetic linkage map construction, set at the default settings with the Kosambi mapping function. Linkage groups were selected at a minimum LOD of 3 for reliable associations. There were 322 polymorphic markers (97 SSRs, 152 DArT, 65 KASP, and eight conserved orthologous set markers) 271 formed linkage groups and 51 remained unlinked giving a total genetic map length of 599 cM.

### Statistical analysis

Analyses of variance (ANOVA) and phenotypic correlations were calculated using the R statistical package [32].

QTL analysis of phenotypic traits was performed using the R/QTL [33] module and the R software suite (vs 2.15.1, R core Team, 2012). QTLs were identified initially by simple interval mapping using both, multiple imputation (imp) and Haley-Knott (hk) algorithm. Genome-wide LOD significance thresholds were calculated by permutation test (1000 repetitions). The significance of QTLs was further tested by fitting a multi-QTL model (fitqtl) by multiple interval mapping and dropping one QTL at a time. Confidence intervals were calculated as 1.5-LOD support intervals (lodint), as intervals in which the LOD score is within 1.5 units of its maximum. Effects with negative signs indicate that the increasing allele is from Weebill, a positive sign designates Bacanora. The variance explained values were estimated from the multi-QTL model.

Genotype x Environment interactions were calculated using the Additive Main effect Multiplicative Interaction method (AMMI) using the R statistical package (R Development Core Team 2008). In this analysis, genotypes displaying the highest levels of phenotypic stability (in this case grain yield) lie closest to the centre of AMMI2 plots. The effect- of individual QTL on yield stability was assessed in the following way:

The distance from the central point of AMMI2 plots for lines carrying a particular allele were summed together.

This process was then repeated for 1000 permutations of randomly selected subsets of the same number of lines.

The effect of the QTL on yield stability was deemed to be significant if the distance from step 1 occurred below the 5% level in step 2.

## Supporting Information

S1 File Climatic conditions, full QTL table, growing environments, and additional data for height and heading data. (DOCX)
