# Supplementary material for: Genetic Dissection of Grain Size and Grain Number Trade-Offs in CIMMYT Wheat Germplasm
Source: PLoS One. 2015 Mar 16;10(3):e0118847. doi: 10.1371/journal.pone.0118847 (PMC4361556; doi:10.1371/journal.pone.0118847)
Supplement: S1 File — (DOCX) [file pone.0118847.s001.docx]

Supplementary information for:

Genetic Dissection of Grain Size and Grain Number Trade-Offs in CIMMYT Wheat Germplasm

Simon Griffiths^1^, Luzie Wingen^1^, Julian Pietragalla^2^, Guillermo Garcia^3^, Ahmed Hasan^3^, Daniel Miralles^3^, Daniel F. Calderini^4^, Jignaben Bipinchandra Ankleshwaria ^1^, Michelle Leverington Waite^1^, James Simmonds^1^, John Snape^1^, Matthew Reynolds^2^

^1^John Innes Centre, Norwich research Park, Norwich, NR4 7UH.

^2^ CIMMYT, Int. Apdo. Postal 6-641, 06600 México, DF, Mexico

^3^Cátedra de Cerealicultura, Departamento de Producción Vegetal, and IFEVA-CONICET, Facultad de Agronomía, Universidad de Buenos Aires, Av. San Martin 4453, C1417DSE Buenos Aires, Argentina

^4^Plant Production and Plant Protection Institute, Universidad Austral de Chile, Campus Isla Teja, Valdivia, Chile

| **Climatic factor** | Growth phase | BA_09 | CF_08 | CF_10 | Ob_07 | Ob_08 | Ob_09 | Ob_10 | Va_08 | Va_09 |
| --- | --- | --- | --- | --- | --- | --- | --- | --- | --- | --- |
| Photoperiod h | LSP | 11.7 | 9.3 | 9.3 | NA | NA | NA | 11.4 | 13.4 | 13.8 |
|  | SEP | 13.2 | 15.1 | 15.1 | NA | NA | NA | 12.1 | 15.1 | 15.5 |
|  | LSP | 12.7 | 7.0 | 5.2 | 14.0 | 15.2 | 14.5 | 15.2 | 10.05 | 9.4 |
| Mean Temp ^o^C | SEP | 13.8 | 10.3 | 9.1 | 13 | 13.2 | 13.2 | 15.6 | 13.1 | 11.1 |
|  | GFP | 18.9 | 15.6 | 16.3 | 15.1 | 15.8 | 15.8 | 17.9 | 16.5 | 14.3 |
|  |  |  |  |  |  |  |  |  |  |  |
| Rainfall mm | LSP | irr | 70.26 | 75.84 | irr | irr | irr | irr | irr | irr |
|  | SEP | irr | 40.7 | 22.2 | irr | irr | irr | irr | irr | irr |
|  | GFP | irr | 54 | 17.85 | irr | irr | irr | irr | irr | irr |
|  |  |  |  |  |  |  |  |  |  |  |
| Incident PAR, MJ m^-2^ d^-1^ | LSP | 5.0 | NA | NA | 7.5 | 7.8 | 7.5 | 7.1 | 9.6 | 7.8 |
|  | SEP | 8.2 | NA | NA | 9.5 | 10.7 | 10.5 | 9.9 | 12.4 | 9.6 |
|  | GFP | 9.5 | NA | NA | 10.6 | 12.1 | 13 | 12.4 | 14.1 | 10.6 |
|  |  |  |  |  |  |  |  |  |  |  |

**Table A.** Climatic condition encountered in Weebill x Bacanora experiments. Photoperiod, mean temperature (temp), rainfall, and incident photosynthetically active radiation (PAR). Crop growth stages are divided into: LSP, the period from seedling emergence to first node detectable; SEP, from first node detectable to anthesis; and GFP, the period from anthesis to physiological maturity. Rainfall for irrigated (irr) experiments was not recorded. PAR data is not available for C F_08 and CF_10.

| **Chr** | **pos** | **LOD** | **%var** | **add eff** | **nr mrk** | **trait** | **env** |
| --- | --- | --- | --- | --- | --- | --- | --- |
| **1A** | **23** | **4.5** | **16.6** | **11.614** | **BS00009444_1A** | **TINBpsqm** | **Ob_09** |
| **1A** | **26** | **3** | **14.4** | **-0.054** | **BS00009559_1A** | **EARBM** | **Ob_08** |
| **1A** | **26** | **3.9** | **15.9** | **7.305** | **BS00009559_1A** | **EARNBpsqm** | **Ob_07** |
| **1A** | **27** | **2** | **8.7** | **12.505** | **BS00009559_1A** | **SPNBpsqm** | **Ob_10** |
| **1A** | **25** | **3.4** | **16.2** | **27.489** | **BS00009559_1A** | **SPNBpsqm** | **BA_09** |
| **1A** | **26** | **4** | **16.2** | **14.584** | **BS00009559_1A** | **SPNBpsqm** | **Ob_07** |
| **1A** | **26** | **2.1** | **8.5** | **-0.11** | **BS00009559_1A** | **TIBM** | **Ob_08** |
| **1A** | **26** | **4.9** | **22.5** | **20.549** | **BS00009559_1A** | **TINBpsqm** | **Ob_07** |
| **1A** | **26** | **3.1** | **12.9** | **0.011** | **wmc312** | **HI** | **CF10** |
| **1A** | **24** | **2.6** | **8.6** | **0.014** | **wmc312** | **HI** | **Ob08** |
| **1A** | **6** | **2.5** | **8.1** | **0.021** | **wPt0527** | **HI** | **Ob09** |
| **1B** | **27** | **5.1** | **17** | **-21.494** | **BS00003743_1A** | **EARNBpsqm** | **Ob_08** |
| **1B** | **27** | **4.7** | **15** | **-21.477** | **BS00003743_1A** | **SPNBpsqm** | **Ob_08** |
| **1B** | **27** | **4.3** | **16** | **-21.394** | **BS00003743_1A** | **TINBpsqm** | **Ob_08** |
| **1B** | **21** | **2.7** | **11.8** | **-989.231** | **BS00004382_1A** | **GRpsqm** | **Ob_09** |
| **1B** | **24** | **2.3** | **10.2** | **1.501** | **BS00011841** | **TGRWT** | **Va_09** |
| **1B** | **29** | **2.1** | **8.2** | **1.363** | **BS00012502_1B** | **TGRWT** | **Ob_08** |
| **1B** | **25** | **2.3** | **11.2** | **0.169** | **cfd48_1B** | **sSPTNBpEAR** | **Ob_09** |
| **1B** | **32** | **4.7** | **17.4** | **1.104** | **gwm274_1B** | **SPBM** | **Ob_07** |
| **1B** | **33** | **2.7** | **11.2** | **0.121** | **gwm274_1B** | **TIBM** | **Ob_08** |
| **1B** | **32** | **2.8** | **9.8** | **0.109** | **gwm274_1B** | **TIBM** | **Ob_09** |
| **1B** | **14** | **3.5** | **16.7** | **-1804.83** | **gwm413_1B** | **GRpsqm** | **Va_09** |
| **1B** | **3.5** | **6.4** | **23.7** | **-0.027** | **wPt1781** | **HI** | **Ob08** |
| **1B** | **20** | **3.8** | **15.8** | **-0.413** | **wPt1781_1B** | **GRYLD** | **Ob_08** |
| **1B** | **57** | **2.3** | **11.3** | **-0.237** | **wPt4532_1B** | **GRYLD** | **Ob_09** |
| **1B** | **13** | **2.3** | **11.5** | **-2.039** | **wPt5281_1B** | **GRNBpSP** | **Va_09** |
| **1B** | **12** | **2.2** | **11.5** | **-1007.56** | **wPt5281_1B** | **GRpsqm** | **CF_08** |
| **1B** | **12** | **2.9** | **12.4** | **-729.032** | **wPt5281_1B** | **GRpsqm** | **Ob_10** |
| **1B** | **11** | **3.2** | **15.3** | **-929.085** | **wPt5281_1B** | **GRpsqm** | **Ob_07** |
| **1B** | **13** | **5.4** | **22.2** | **-1628.55** | **wPt5281_1B** | **GRpsqm** | **Ob_08** |
| **1B** | **8** | **2.4** | **10.3** | **-13.202** | **wPt5281_1B** | **SPNBpsqm** | **Ob_10** |
| **1B** | **12** | **4.1** | **20.5** | **0.344** | **wPt5281_1B** | **sSPTNBpEAR** | **CF_10** |
| **1B** | **9** | **2.2** | **7.8** | **1** | **wPt5281_1B** | **TGRWT** | **Ob_10** |
| **1B** | **9** | **3.2** | **12.8** | **2.024** | **wPt5281_1B** | **TGRWT** | **BA_09** |
| **1B** | **44** | **3.2** | **13.4** | **-0.011** | **wPt9809** | **HI** | **CF10** |
| **1B** | **44** | **5.5** | **19.2** | **-0.021** | **wPt9809** | **HI** | **Ob09** |
| **1B** | **45** | **2.7** | **13.8** | **-930.226** | **wPt9809_1B** | **GRpsqm** | **CF_10** |
| **1B** | **43** | **2.9** | **12.5** | **1.382** | **wPt9809_1B** | **TGRWT** | **Ob_07** |
| **1B** | **24** | **1** | **3** | **2.334** | **wPt9857.a** | **DTAD** | **Ob07** |
| **1B** | **25** | **1.1** | **4** | **1.759** | **wPt9857.a** | **DTAD** | **Ob08** |
| **1Db** | **15** | **2** | **7.7** | **0.39** | **cfd48_1D** | **EARLG** | **Ob_08** |
| **2Aa** | **0** | **2.2** | **11.4** | **0.124** | **wPt1499_2A** | **GRpSPT** | **CF_10** |
| **2Ab** | **55** | **2.4** | **14.8** | **0.099** | **gwm526_2A** | **GRNBpSPT** | **BA_09** |
| **2Ab** | **3** | **4.1** | **20.3** | **-29.657** | **wPt7626_2A** | **SPNBpsqm** | **BA_09** |
| **2Bb** | **13** | **3.4** | **14.3** | **0.559** | **wPt8569_2B** | **SPTNBpEAR** | **Ob_08** |
| **2D** | **24** | **2.2** | **11.7** | **0.296** | **wPt0298_2D** | **SPTNBpEAR** | **CF_10** |
| **2D** | **0** | **2.1** | **10.3** | **0.011** | **wPt1554_2D** | **HI** | **Va_09** |
| **3Aa** | **25** | **2** | **8.6** | **-0.247** | **wPt3278_3A** | **EARLG** | **CF_10** |
| **3Aa** | **6** | **2.7** | **13.9** | **-0.077** | **wPt4407_3A** | **DTMA** | **CF_10** |
| **3Aa** | **18** | **2.3** | **8.6** | **1.867** | **wPt7608_3A** | **Ht** | **Va_09** |
| **4A** | **1** | **2.5** | **8.1** | **-0.015** | **wmc048a.b** | **HI** | **Ob09** |
| **4A** | **0** | **2.9** | **17.5** | **2.397** | **wmc48a_4a** | **Ht** | **BA_09** |
| **4A** | **0** | **3.2** | **12.1** | **2.138** | **wmc48a_4a** | **Ht** | **Va_09** |
| **4A** | **0** | **5** | **22.9** | **2.241** | **wmc48a_4a** | **Ht** | **Va_08** |
| **4A** | **0** | **2.8** | **9.8** | **0.107** | **wmc48a_4a** | **TIBM** | **Ob_09** |
| **4A** | **6** | **2.5** | **7.8** | **-14.685** | **wmc89_4a** | **EARNBpsqm** | **Ob_08** |
| **4A** | **6** | **2.9** | **9.1** | **-15.01** | **wmc89_4a** | **SPNBpsqm** | **Ob_08** |
| **4A** | **6** | **2.2** | **7.8** | **-15.219** | **wmc89_4a** | **TINBpsqm** | **Ob_08** |
| **4A** | **65** | **2.7** | **13.7** | **0.421** | **wPt6997_4A** | **SPTNBpEAR** | **BA_09** |
| **4A** | **71** | **2.5** | **12.4** | **-1.883** | **wPt7924_4A** | **Ht** | **Ob_09** |
| **5Aa** | **14** | **2.1** | **10.2** | **-18.721** | **wPt3620_5A** | **EARBMpsqm** | **Ob_08** |
| **5Ab** | **41** | **2.3** | **9** | **-0.307** | **wPt0373_5A** | **EARLG** | **Ob_09** |
| **5Ab** | **40** | **2.8** | **11.5** | **-0.307** | **wPt9834_5A** | **EARLG** | **Ob_07** |
| **5Ab** | **32** | **3.1** | **13.2** | **-0.582** | **wPt9834_5A** | **SPTNBpEAR** | **Ob_07** |
| **5B** | **90** | **3.3** | **16** | **-0.099** | **BS0003615_5B** | **GRNBpSPT** | **Ob_10** |
| **5B** | **94** | **2.4** | **11.9** | **1.198** | **vrnB1_5B** | **DTAD** | **Va_08** |
| **5B** | **94** | **2.6** | **12.9** | **1.003** | **vrnB1_5B** | **DTAD** | **Va_09** |
| **5B** | **94** | **7.1** | **27.4** | **3.002** | **vrnB1_5B** | **DTAD** | **Ob_08** |
| **5B** | **94** | **11.6** | **40.5** | **4.981** | **vrnB1_5B** | **DTAD** | **Ob_07** |
| **5B** | **94** | **12.1** | **46.8** | **6.315** | **vrnB1_5B** | **DTAD** | **Ob_09** |
| **5B** | **56.8** | **9.3** | **34.2** | **4.718** | **vrnB1_5B** | **DTAD** | **Ob07** |
| **5B** | **56.8** | **6.1** | **24.5** | **2.774** | **vrnB1_5B** | **DTAD** | **Ob08** |
| **5B** | **56.8** | **11.291** | **44.616** | **6.122** | **vrnB1_5B** | **DTAD** | **Ob09** |
| **5B** | **53** | **2.33** | **11.48** | **1.086** | **vrnB1_5B** | **DTAD** | **Va08** |
| **5B** | **94** | **10.3** | **41.6** | **5.096** | **vrnB1_5B** | **DTEM** | **Ob_09** |
| **5B** | **94** | **10.9** | **38.5** | **5.02** | **vrnB1_5B** | **DTEM** | **Ob_07** |
| **5B** | **94** | **3.9** | **18.4** | **1.709** | **vrnB1_5B** | **DTMA** | **Ob_08** |
| **5B** | **94** | **7.1** | **30.9** | **3.164** | **vrnB1_5B** | **DTMA** | **Ob_09** |
| **5B** | **94** | **7.6** | **32.9** | **2.072** | **vrnB1_5B** | **DTMA** | **Ob_07** |
| **5B** | **96** | **4.3** | **17.7** | **0.103** | **vrnB1_5B** | **EARBM** | **Ob_09** |
| **5B** | **94** | **4.3** | **20.2** | **1.503** | **vrnB1_5B** | **GFR** | **Ob_09** |
| **5B** | **94** | **2.6** | **9.3** | **0.93** | **vrnB1_5B** | **SPBM** | **Ob_07** |
| **5B** | **94** | **6.2** | **27.8** | **1.146** | **vrnB1_5B** | **SPTNBpEAR** | **Ob_09** |
| **5B** | **87** | **3** | **15.2** | **-0.451** | **wPt8054_5B** | **SPTNBpEAR** | **BA_09** |
| **5B** | **8** | **2.7** | **10.8** | **-6.236** | **wPt9800_5B** | **EARNBpsqm** | **Ob_07** |
| **5B** | **2** | **2.4** | **7.3** | **-17.588** | **wPt9800_5B** | **SPNBpsqm** | **Ob_08** |
| **5B** | **7** | **2.9** | **11.3** | **-10.997** | **wPt9800_5B** | **SPNBpsqm** | **Ob_07** |
| **5B** | **3** | **2.2** | **7.9** | **-17.02** | **wPt9800_5B** | **TINBpsqm** | **Ob_08** |
| **6A** | **28** | **2.2** | **10** | **1.407** | **BS00018855** | **Ht** | **Ob_10** |
| **7Aa** | **4** | **3** | **11** | **1.102** | **wPt4744_7A** | **TGRWT** | **Ob_10** |
| **7B** | **23** | **2.4** | **11.6** | **-0.236** | **BS00011065** | **GRYLD** | **Ob_07** |
| **7B** | **29** | **2.7** | **10.7** | **-0.384** | **gwm577_7B** | **EARLG** | **Ob_08** |
| **7B** | **28** | **3.9** | **16.7** | **-0.41** | **gwm577_7B** | **EARLG** | **Ob_07** |
| **7B** | **29** | **4.7** | **19.8** | **-0.465** | **gwm577_7B** | **EARLG** | **Ob_09** |
| **7B** | **34** | **2** | **12.6** | **-0.055** | **gwm577_7B** | **GFR** | **BA_09** |
| **7B** | **32** | **2.4** | **11.6** | **-0.028** | **gwm577_7B** | **GFR** | **Ob_10** |
| **7B** | **34** | **3.2** | **13.6** | **-1.557** | **gwm577_7B** | **TGRWT** | **Ob_07** |
| **7B** | **33** | **3.4** | **13.8** | **-1.454** | **gwm577_7B** | **TGRWT** | **Ob_08** |
| **7B** | **33** | **3.6** | **13.5** | **-1.453** | **gwm577_7B** | **TGRWT** | **Ob_10** |
| **7B** | **34** | **5.2** | **22.1** | **-2.36** | **gwm577_7B** | **TGRWT** | **BA_09** |
| **7B** | **0** | **2.2** | **8.9** | **0.49** | **wmc517_7b** | **SPTNBpEAR** | **Ob_07** |
| **7B** | **38** | **4.7** | **22.7** | **-0.587** | **wPt8007_7B** | **GRYLD** | **BA_09** |
| **7B** | **40** | **4.5** | **26** | **-0.015** | **wPt8007_7B** | **HI** | **BA_09** |
| **7B** | **36** | **2.8** | **16.8** | **-79.847** | **wPt8007_7B** | **PLBM** | **BA_09** |
| **7B** | **39** | **2.8** | **12.6** | **-1.599** | **wPt8007_7B** | **TGRWT** | **CF_08** |
| **7B** | **39** | **5.1** | **18.7** | **-1.654** | **wPt8007_7B** | **TGRWT** | **CF_10** |
| **7B** | **3** | **5.1** | **23.3** | **-1.604** | **wPt9925_7B** | **TGRWT** | **Ob_09** |
| **7Bb** | **50** | **4.668** | **26.77** | **-0.015** | **wPt4038** | **HI** | **BA09** |

**Table B.** QTL with a LOD score greater than 2 identified in the Weebill x Bacanora population. Chromosomes broken into multiple linkage groups carry a single letter suffix. Column heading abbreviations: Chr, chromosome; pos, centiMorgan position on chromosome; LOD, LOD score; %var, percentage of phenotypic variation explained; add eff, additive effect; nr mrk, nearest marker to QTL peak; nr mrk pos, centiMorgan position of nearest marker; CIstart, CentiMorgan position of one side of the QTL confidence interval, CIend, centiMorgan position of the other side of the QTL confidence interval; st mrk, molecular marker on side of QTL confidence interval; end mrk, molecular marker on other side of QTL confidence interval; trait, trait short name, full names shown in Table 2; env, environment in which QTL was detected, full names shown in Supplementary Table 3.

| Location | Environments | Design | Latitude | Longitude | Altitude | Sowing date | Seed rate m-^2^ |
| --- | --- | --- | --- | --- | --- | --- | --- |
| Buenos Aires (ARG) | BA_09 | Complete randomised | 34°35'S | 58°29'W | 26 | July 5^th^ 2009 | 250 |
| Norwich (UK) | CF_08 |  | 52°37’N | 1°10’E | 31 | October 31^st^ 2007 | 250 |
|  | CF_10 | Randomised block | 52°37’N | 1°10’E | 37 | October 30^th^ 2009 | 250 |
| Cdad. Obregon (MEX) | Ob_07 |  | 27°25'N | 109°54'W | 38 |  | 180 |
|  | Ob_08 | Alpha lattice | 27°25'N | 109°54'W | 38 |  |  |
|  | Ob_09 |  | 27°25'N | 109°54'W | 38 |  |  |
|  | Ob_10 |  | 27°25'N | 109°54'W | 38 | December 2^nd^ 2009 | 180 |
| Valdivia (CHI) | Va_08 | Randomised block | 39°47’S | 73°14’W | 19 | Aug 23^rd^ 2008 | 350 |
|  | Va_09 |  | 39°47’S | 73°14’W | 19 | Sep 4^th^ 2009 | 350 |

**Table C.** Details of environments in which the WxB population was grown. Two replicates were grown in Obregon, three in all other environments. Climatic conditions for these sites can be found in Supplementary Table 1.

| Trait | Env | | Weebill | | Bacanora | WxB mean | WxB sd | WxB CV | WxB her.block | WxB adj.r.sq | WxB Pr..F. | WxB signif |
| --- | --- | --- | --- | --- | --- | --- | --- | --- | --- | --- | --- | --- |
| DTAD | BA_09 | | 87.7 | | 88 | 88.1 | 3.7 | 4.2 | 0.88 | 0.72 | 1.42e-32 | *** |
|  | Ob_07 | | 82.5 | | 80.5 | 87.2 | 8.16 | 9.4 | 0.98 | 0.94 | 6.29e-51 | *** |
|  | Ob_08 | | 89 | | 87.5 | 90.8 | 5.45 | 6 | 0.97 | 0.93 | 1.58e-48 | *** |
|  | Ob_09 | | 78.5 | | 74 | 85.8 | 9.94 | 11.6 | 0.95 | 0.89 | 1.40e-38 | *** |
|  | Ob_10 | | 83 | | 83 | 66.1 | 4.58 | 6.9 | 0.94 | 0.88 | 3.57e-37 | *** |
|  | Va_09 | | 79.7 | | 74.7 | 78.5 | 3.47 | 4.4 | 1 | 0.99 | 3.3e-185 | *** |
|  | Va_10 | | 90.3 | | 86.6 | 86.3 | 2.79 | 3.2 | 0.99 | 0.98 | 1.56e-155 | *** |
| DTEM | CF_08 | | 20.3 | | 23 | 21.7 | 3.61 | 16.6 | 0.59 | 0.32 | 5.34e-08 | *** |
|  | CF_10 | | 32.3 | | 28.6 | 31.7 | 2.88 | 9.09 | 0.64 | 0.38 | 2.16e-10 | *** |
|  | Ob_07 | | 51.5 | | 49.5 | 63.8 | 8.27 | 13 | 0.98 | 0.94 | 2.70e-51 | *** |
|  | Ob_09 | | 84 | | 79 | 79.2 | 8.45 | 10.7 | 0.94 | 0.88 | 2.71e-36 | *** |
| HT | BA_09 | | 80.6 | | 69.3 | 75.9 | 6.46 | 8.5 | 0.78 | 0.55 | 2e-16 | *** |
|  | CF_10 | | 76 | | 61.3 | 69.7 | 7.09 | 10.2 | 0.88 | 0.72 | 1.58e-38 | *** |
|  | Ob_07 | | 101.5 | | 110 | 97.2 | 7.5 | 7.7 | 0.97 | 0.79 | 5.73e-24 | *** |
|  | Ob_08 | | 106.5 | | 97.9 | 100.4 | 6.39 | 6.4 | 0.83 | 0.7 | 3.83e-18 | *** |
|  | Ob_09 | | 107.5 | | 87 | 97.2 | 6.61 | 6.8 | 0.89 | 0.79 | 2.03e-24 | *** |
|  | Ob_10 | | 87.8 | | 78.7 | 83.6 | 4.71 | 5.6 | 0.76 | 0.62 | 1.2e-12 | *** |
|  | Va_09 | | 83.3 | | 74.7 | 82.4 | 5.7 | 6.9 | 0.91 | 0.78 | 1.65e-49 | *** |
|  | Va_10 | | 80.7 | | 72.2 | 76.2 | 6.2 | 8.1 | 0.96 | 0.89 | 1.68e-80 | *** |
|  |  |  | |  |  |  |  |  |  |  |  |  |
|  |  |  | |  |  |  |  |  |  |  |  |  |

**Table D.** Weebill, Bacanora, and WxB population ANOVA and heritabilities . Full trait names are given in Table 2 of main text. Abbreviations are: Mean, unadjusted arithmetic mean; var, variance; sd, standard deviation; CV, coefficient of variation; her. Block; heritability estimate; r.sq; R-squared; adj.r.sq, adjusted R squared; Pr.F, p value of F test; signif, significance level (***0.0001, ** 0.001, * 0.05; F, F statistic; df, degrees of freedom; df.res; df in the residuals; sum.sq; sums of square for trait; sum.sq.res, sums of square for residuals; mean.sq, means of square for trait; mean.sq.res, means of square for residuals; anovamean, mean from the linear model (or anova); ranef.se, standard errors of random effects; site, site; year. Yield components are shown in main text.
